# Dexamethasone protection from TNF-alpha-induced cell death in MCF-7 cells requires NF-kappaB and is independent from AKT

**DOI:** 10.1186/1471-2121-7-9

**Published:** 2006-02-21

**Authors:** Catalina Machuca, Criselda Mendoza-Milla, Emilio Córdova, Salvador Mejía, Luis Covarrubias, José Ventura, Alejandro Zentella

**Affiliations:** 1 Carrera de Biología, Facultad de Estudios Superiores ZARAGOZA, UNAM. C.P. 09230, México D.F., México; 2 Instituto Nacional de Enfermedades Respiratorias, Secretaría de Salud, C.P. 14000, México D.F., México; 3 Departamento de Medicina Genómica y Toxicología Ambiental, Instituto de Investigaciones Biomédicas, UNAM. C.P. 04510, México D.F., México; 4 Departamento de Bioquímica, Instituto Nacional de Ciencias Médicas y Nutrición "Salvador Zubirán", Secretaría de Salud, C.P. 14000, México, D.F., México; 5 Departamento de Genética del Desarrollo y Fisiología Molecular, Instituto de Biotecnología, UNAM. C.P. 62210, Cuernavaca Mor. México

## Abstract

**Background:**

The biochemical bases for hormone dependence in breast cancer have been recognized as an important element in tumor resistance, proliferation and metastasis. On this respect, dexamethasone (Dex) dependent protection against TNF-alpha-mediated cell death in the MCF-7 cell line has been demonstrated to be a useful model for the study of this type of cancer. Recently, cytoplasmic signaling induced by steroid receptors has been described, such as the activation of the PI3K/Akt and NF-kappaB pathways. We evaluated their possible participation in the Dex-dependent protection against TNF-alpha-mediated cell death.

**Results:**

Cellular cultures of the MCF-7 cell line were exposed to either, TNF-alpha or TNF-alpha and Dex, and cell viability was evaluated. Next, negative dominants of PI3K and IkappaB-alpha, designed to block the PI3K/Akt and NF-kappaB pathways, respectively, were transfected and selection and evaluation of several clones overexpressing the mutants were examined. Also, correlation with inhibitor of apoptosis proteins (IAPs) expression was examined. Independent inhibition of these two pathways allowed us to test their participation in Dex-dependent protection against TNF-alpha-cytotoxicity in MCF-7 cells. Expression of the PI3K dominant negative mutant did not alter the protection conferred by Dex against TNF-alpha mediated cell death. Contrariwise, clones expressing the IkappaB-alpha dominant negative mutant lost the Dex-conferred protection against TNF-alpha. In these clones degradation of c-IAP was accelerated, while that of XIAP was remained unaffected.

**Conclusion:**

NF-kappaB, but not PI3K/Akt activation, is required for the Dex protective effect against TNF-alpha-mediated cell death, and correlates with lack of degradation of the anti-apoptotic protein c-IAP1.

## Background

Breast cancer is one of the most important oncologic diseases worldwide, and in Mexico is the second most frequent neoplasia in women population [1]. It is widely accepted that among the factors involved in the development of this ailment are long-standing inflammation and steroid hormone regulation. On this respect, the pro-inflammatory cytokine tumor necrosis factor alpha (TNF-α) has been postulated as a key player in the tumor microenvironment, but has a paradoxical role in disease evolution: It can act both as a necrotic or as a promoting factor [2] e.g., the endogenous TNF-α chronically produced in the tumor microenvironment enhances both tumor development and spreading, while local administration of high-doses of TNF-α is antiangiogenic and has a powerful anti-tumoral effect [3]. It is worth to note that TNF-α acts as a mediator of the apoptotic process and has selective cytotoxicity against malignant breast tumor cells, promoting an apoptotic type of cell death in MCF-7 cells [4].

TNF-α, a 17,000 kDa polypeptide, elicits a wide range of biological responses, including inflammation, cell proliferation, differentiation and apoptosis [5]. The binding of TNF-α to the TNF receptor type I (TNF-RI) promotes the recruitment of several intracellular adaptors which in turn, activate multiple signal transduction pathways [6]. While recruitment of death domain (DD) containing adaptors such as Fas associated DD (FADD) and TNF-R associated DD (TRADD) can lead to the activation of signal transduction pathways that induce apoptosis, recruitment of TNF-RI associated factors (TRAFs) can lead to the activation of multiple cell survival intracellular signals such as NF-κB, JNK, p38 and Erk [7].

Glucocorticoids (GCs) are essential steroid hormones required for the maintenance of several key physiological and developmental processes. GCs act through binding to the GC receptor (GR), which is followed by GR translocation into the nucleus and trans-activation or trans-repression of target genes [8]. In addition, rapid nongenomic effects of GCs have been described [9]. There is a dual and cell-type-specific role for GCs in cell death regulation: GCs are able to induce apoptosis in lymphocytes, leukemic cells, lymphomas and multiple myeloma cells (reviewed in 10). However, in other cell types such as hepatocytes [11], vascular endothelial cells [12], osteoclasts [13] and particularly in mammary epithelial cells [14], GCs can inhibit apoptosis induced by a variety of different stimuli. Besides, in the human breast tumor derived cell line MCF-7 the synthetic GC dexamethasone (Dex) is able to completely abrogate the TNF-α-mediated cell death [15,16]. In fact, this system has been recognized as a valuable experimental model to study hormone dependent breast cancer cells. However, and despite many efforts, the mechanism used by Dex to interfere with the TNF-α-dependent cell death remains poorly understood.

To investigate the interaction of TNF-α and Dex we used the MCF-7 cell line and evaluated the contribution of two main routes involved in cell survival: the nuclear factor κB (NF-κB) and the phosphatidyl inositol 3 kinase (PI3K) activated pathways. NF-κB is a heterodimer, typically consisting of the p50 and p65 monomers, sequestered in the cytoplasm of most un-stimulated cells by members of the family of inhibitory proteins IκB [17]. NF-κB is activated by TNF-α through ubiquitin-mediated degradation of IκBs [18]. After IκB degradation, NF-κB translocates to the nucleus and binds to κB sites up-regulating a panel of proteins, including the anti-apoptotic proteins (IAP) c-IAP1, c-IAP2 and XIAP [19]. Deficiencies in NF-κB activation or interference with the synthesis of new proteins render a cell extremely sensitive to TNF-α induced apoptosis [20].

In addition to their participation in survival and proliferation, PI3K and its target PKB/Akt, have emerged as critical signaling molecules that regulate multiple cellular processes [21]. The ability of PI3K or Akt to suppress apoptosis has been attributed to both, Bad and caspase-9 phosphorylation [22], as well as ceramide regulation [23]. In addition to these anti-apoptotic effects, Akt can also contribute activating NIK, with the consequent nuclear translocation of NF-κB [24]. Thus, depending on cell context and cell type, TNF-α is able to induce cell survival or apoptosis pathways.

It has been demonstrated that NF-κB is able to inhibit apoptosis triggered by TNF-α, and that NF-κB activation by both, constitutively active PI3K or Akt, suppresses TNF-α-dependent apoptosis in MCF-7 and HEK 293 cells [25]. We found that Dex protection against a TNF-α-dependent cell death was not affected by the expression of a dominant-negative PI3K mutant protein (Δp85). However, expression of a non-degradable IκBα mutant protein (dnIKBα) completely abrogated Dex protection against TNF-α-induced cell death. In addition, expression of dnIκBα was accompanied by downregulation of the anti-apoptotic protein c-IAP1.

## Results

### Dexamethasone blocks the cytotoxicity of TNF-α in the breast carcinoma-derived cell line MCF-7

**Figure 1:**
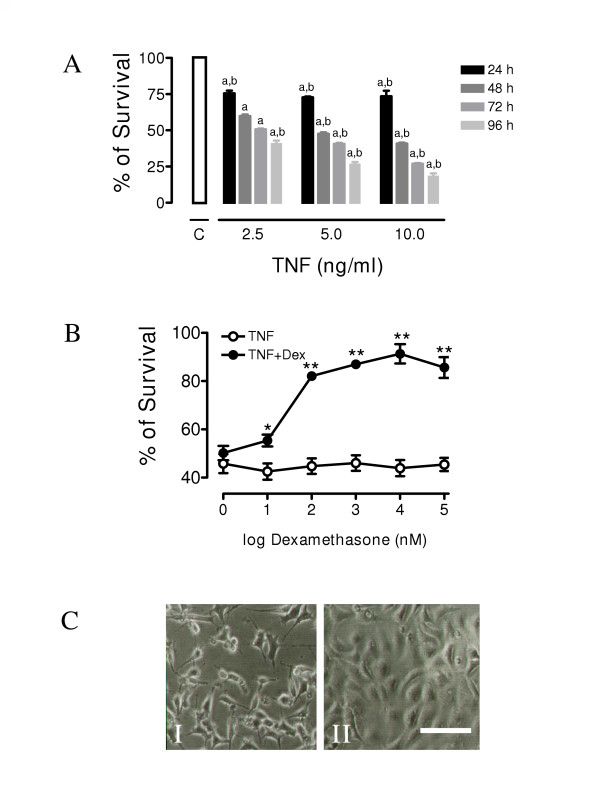
**Dexamethasone inhibits in a dose-dependent manner the TNF-α mediated cytotoxicity in MCF-7 cells: **(**A**) MCF-7 cells were treated with different concentrations of TNF-α and cell survival was determined at the indicated times (24, 48, 72 and 96 h). Values are mean ± SD from three independent experiments performed in triplicate. **a **indicates p < 0.001 with respect to control value. **b **indicates p < 0.001 with respect to the other values of the same group: (**B**) Cell survival of cultures treated with TNF-α (10 ng ml-^1^) for 48 h in the presence of different concentrations of Dex. Values are mean ± SD from three independent experiments performed in triplicate. * indicates p < 0.05 vs TNF + vehicle, ** indicates p < 0.001 vs. TNF + vehicle.: (**C**) Micrograph of sub-confluent MCF-7 cells treated for 48 h with 10 ng ml^-1 ^of TNF-α in the presence (right panel) or absence of Dex 10 μM (left panel) (Scale bar: 25 μm).

In order to determine the sensitivity of MCF-7 cells to TNF-α cell cultures were incubated with 2, 5 and 10 ng ml^-1 ^of TNF-α for different periods of time. Cell survival was determined by crystal violet assay (Figure 1A). Like it was reported before [16], TNF-α showed a dose- and time-dependent cytotoxic effect on cell survival. We observed that the minimum cell survival of 17.4 % was at the highest dose of TNF-α used (10 ng ml^-1^) at the longest period of incubation (96 h). The maximum change in cytotoxicity occurred between 24 and 48 h with about 80% to 45% survival respectively with a dose of 10 ng ml^-1^. At longer periods of time cell mortality increased even further (Figure 1A). To evaluate the range of the protective doses of Dex against TNF-α cytotoxicity, MCF-7 cells were co-incubated with TNF-α (10 ng ml^-1^) at different concentrations of Dex or its vehicle for 48 h (Figure 1B). Dex protected cells against TNF-α-induced cell death in a dose-dependent manner. 10 μM Dex offered a complete protection against the cytotoxic effect of TNF-α and, at 100 μM, had a toxic effect on its own. In subsecuent experiments we used a concentration of Dex of 10 μM. As shown in figure 1C, Dex 10 μM was able to prevent the TNF-α-dependent reduction in cell number in MCF-7 cultures without any alteration of cellular morphology even after 48 h of incubation. To elucidate the molecular mechanism involved in the protective effects of Dex against TNF-α dependent cell death, we evaluated the possible participation of the PI3K and NF-κB survival pathways.

### Dexamethasone protection from TNF-α cytotoxicity is not mediated by the PI3K/Akt pathway

**Figure 2:**
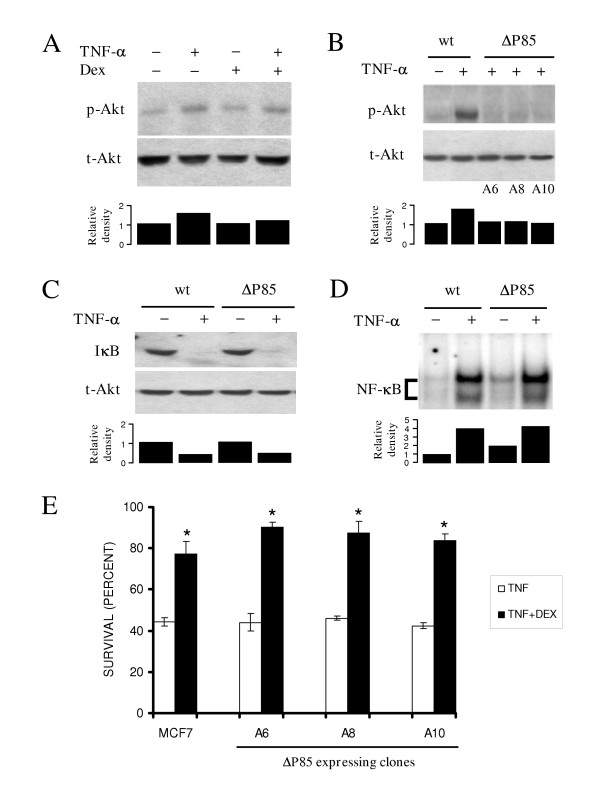
**The PI3K/Akt pathway does not participate in the dexamethasone-mediated protection from TNF-α-dependent cell death in MCF-7 cells: **(**A**) Cell cultures were treated with TNF-α (10 ng ml^-1^), dexamethasone (Dex) (10 μM) or both for 20 min: Western blotts of whole-cell extracts were performed with anti-phosphorylated Akt (pAkt) specific antibodies (upper panel): After stripping, membranes were re-blotted against total Akt (tAkt) (lower panel): (**B**) Parental cells and cells from ΔP85-expressing MCF-7 clones (A6, A8 and A10) were treated with TNF-α (10 ng ml-^1^) for 20 min and total (tAkt) and phosphorylated (pAkt) Akt were determined as in A: IκB protein degradation (**C**) and NF-κB nuclear translocation (**D**) were determined by Western blot and EMSA respectively in parental and ΔP85 expressing cell clone A6 treated with TNF-α (10 ng ml^-1^) for 20 min. Each blot is representative of three independent experiments. Below the blots in **A**, **B**, **C**, and **D **the bar graphs indicate the relative density of each lane with respect to control, which has an arbitrary value of 1: (**E**) Dexamethasone protection against TNF-α-mediated cytotoxicity was evaluated in parental MCF-7 cells (MCF-7) and three independent clones expressing the ΔP85 protein (A6, A8 and A10): Cell viability of clones incubated either with TNF-α (10 ng ml^-1^) alone or with TNF-α and Dex (10 μM) for 48 hrs was determined. Values are mean ± SD from three independent experiments performed in triplicate. * indicates p < 0.01 vs TNF.

Activation of Akt is a phosphorylation dependent event mediated by a PI3K dependent kinase (PDK) that occurs in response to different extracellular stimuli. As is shown in figure 2A, TNF-α stimulation of MCF-7 cells resulted in an increase of the phosphorylated state of Akt (pAkt). Dex treatment did not affect the levels of pAkt in unstimulated or TNF-α treated cells (Figure 2A), suggesting that Akt phosphorylation does not participate in Dex protection. Expression of the PI3K dominant negative mutant (ΔP85) protein in three independent clones, A6, A8 and A10, abrogated the TNF-α-associated phosphorylation of Akt (Figure 2B, upper panel) without affecting total Akt protein levels (Figure 2B, lower panel). It is worth to note that Akt activation has been reported to play an important role in IκB degradation and NF-κB activation in diverse cell types [26,27]. To assure that PI3K/Akt signal pathway inhibition did not affect the NF-κB survival route, we tested if overexpression of ΔP85 could alter the IκB degradation and NF-κB nuclear translocation. Figure 2C shows that the inhibition of Akt phosphorylation by ΔP85 in the clone 6 did not affect the IκB degradation induced by TNF-α stimulation. Moreover, the NF-κB activation induced by TNF-α was not affected in these cells (Figure 2D). Taken together, these results showed that, despite the fact that the ΔP85 mutant protein was able to interfere with Akt activation this did not affect the TNF-α-dependent NF-κB activation.

Finally, we evaluated the survival of cells from the three different clones expressing the ΔP85 mutant protein after TNF-α and Dex treatment. As shown in figure 2E, no statistical difference was observed in the protection conferred by Dex in any one of the three clones when compared to the parental cell line. Dex-dependent protection against TNF-α remained unchanged in cells transfected with the empty vector (data not shown). These results suggest that the PI3K/Akt pathway is not involved in the Dex protection against TNF-α cytotoxicity.

### NF-κB participates in the dexamethasone protection from a TNF-α mediated cell death

**Figure 3:**
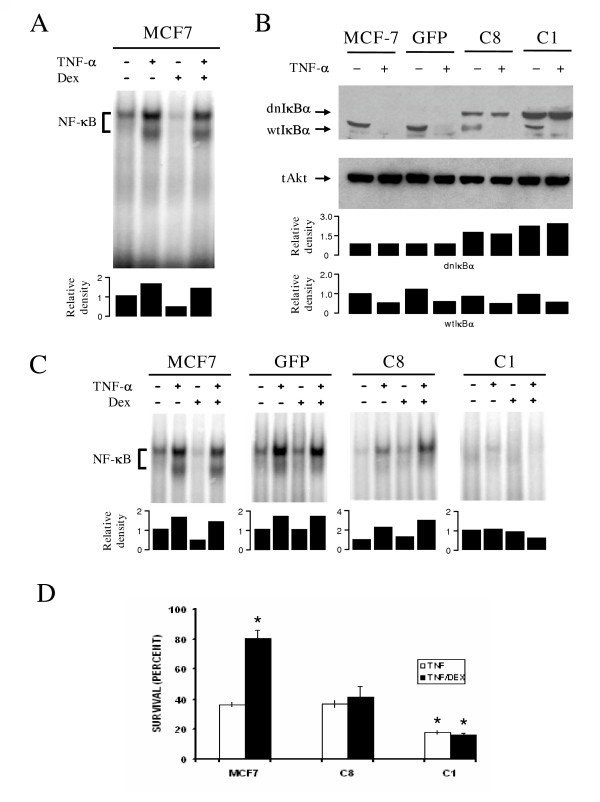
**Inhibition of NF-κB activity abrogates the dexamethasone protection from TNF-α mediated cell death: **(**A**) MCF-7 cells were treated as in figure 2A and DNA NF-κB binding was evaluated in nuclear extracts by EMSA: (**B**) Wild type MCF-7 cells and MCF-7 clones 1 and 8 expressing a dominant negative IκB protein (dnIκB) or the empty vector (GFP) were treated with TNF-α (10 ng ml^-1^) for 20 min: After cell lysis, endogenous (wtIκB) and recombinant (dnIκB) proteins were detected in whole cell extract by Western blot: Total Akt (tAkt) is shown as loading control: (**C**) Indicated cell cultures were treated as in 2A: Then, NF-κB nuclear translocation was evaluated by EMSA. Each blot is representative of three independent experiments. Below the blots in **A**, **B**, and **C **the bar graphs indicate the relative density of each lane with respect to control, which has an arbitrary value of 1. In C the first bar graph represents the relative densities for dnIκBα, while the second bar graph represents the relative densities for wtIκBα: (**D**) The indicated cell clones were incubated with TNF-α (10 ng ml^-1^) alone or TNF-α (10 ng ml^-1^) and Dex (10 μM) for 48 hrs and cell viability was determined. Values are mean ± SD from three independent experiments performed in triplicate. * indicates p < 0.01 with respect to MCF-7 survival after TNF-α treatment.

Our next goal was to determine the role of NF-κB in the protection conferred by Dex analyzing by EMSA the NF-κB nuclear translocation after Dex treatment in the absence or presence of TNF-α. TNF-α treatment showed two DNA/NF-κB complexes (Figure 3A); supershift analysis with anti p65 and p50 antibodies revealed the presence of p65 in both of them, whereas p50 was present only in the complex with lower mobility (data not shown). On its own, Dex treatment led to a slight decrease in the NF-kB signal (Figure 3A, lane 3), but it had no effect on the two NF-κB complexes induced by TNF-α. Taken together, these results indicate that Dex protection does not affect NF-κB activation.

To further analyze the participation of NF-κB in Dex protection, a non-degradable recombinant IκBα mutant protein (dnIκBα) was transfected into MCF-7 cells. Endogenous (wtIκBα) and recombinant (dnIκBα) forms of IκBα were clearly distinguished due to the presence of a TAG sequence in the mutant form resulting in a higher molecular weight protein. As expected, wtIκBα was degraded in all clones when stimulated with TNF-α while dnIκBα, lacking the two serine phosphorylation sites, was not degraded after TNF-α stimulation (Figure 3B). When the clone C1, with a high level of expression of dnIkBα, was treated with TNF-α, NF-κB translocation was significantly reduced both in the presence or absence of Dex (Figure 3C). In contrast, when the clone C8, expressing low levels of dnIκBα, was stimulated with TNF-α, NF-κB activation was almost as intense as in the parental MCF-7 cells or in those transfected with the empty vector (Figure 3C, compare lines 9–12 with lanes 1–4 and 5–8). When cell survival was determined in the different clones after exposure to TNF-α in the presence or absence of Dex, we found that Dex protection was completely abrogated in the clone expressing high levels of the dnIκBα (Figure 3D). In addition, the clone C1 became more susceptible to the cytotoxic effect of TNF-α since cell viability fell by half when compared to the clone C8 or parental untransfected cells. In both clones Dex protection was dramatically reduced when compared to the parental cell line: viability was indistinguishable from that of cells exposed only to TNF-α (Figure 3D). These results suggest a dose-dependent effect of active NF-κB in the protection mediated by Dex.

### Loss of dexamethasone protection in dnIκBα expressing cells correlates with a lower c-IAP1 content

**Figure 4:**
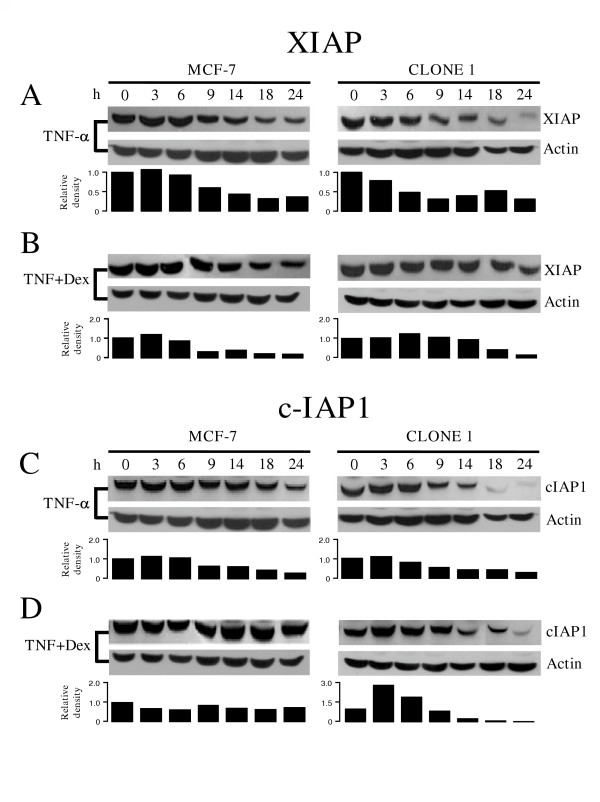
**The dominant negative IκB mutant protein stimulates the dexamethasone induced downregulation of IAPs: **MCF-7 parental cells and Clone 1 of the dnIκB expressing cells were treated for 20 min with TNF-α (10 ng ml^-1^) alone (A and C) or in combination with Dex (10 μM) (B and D): Protein expression of XIAP (A and B) or c-IAP (C and D) at different times was determined by Western blot: Actin was used as loading control. Each blot is representative of three independent experiments. Below the blots the bar graphs indicate the relative density of each lane with respect to the respective control, which has an arbitrary value of 1.

As has been previously reported [16], Dex protection against TNF-α cytotoxicity correlates with inhibition of XIAP and c-IAP1 protein degradation. TNF-α exposure resulted in a time dependent decline of both XIAP and c-IAP1 in the parental MCF-7 cells (left panels, figures 4A and 4C). Dex treatment alone had no effect on the levels of either of the two antiapoptotic proteins even after 24 hrs (data not shown). Simultaneous stimulation with TNF-α and Dex led to higher levels of the two proteins when compared to the effect of TNF-α alone (Figure 4B and 4D, left panels), as previously shown [16]. When MCF-7 cells expressing dnIκBα were treated with TNF-α (Figure 4A and 4C, right panels) the downregulation of XIAP and, especially that of c-IAP1, was accelerated and resulted in lower levels than those reached in parental cells. Treatment with TNF-α plus Dex led to a differential effect in the two antiapoptotic proteins: In the case of XIAP, the protein downregulation time course was similar to that of the parental cells (Figure 4B, right panel), while in the case of c-IAP1 the protein downregulation was stimulated in response to Dex (Figure 4C and 4D, right panels). These results indicate that only cIAP1 levels correlate with the protection conferred by Dex and with NF-κB activation.

## Discussion

To contribute to the understanding of cancer genesis, the breast cancer derived cell line MCF-7 has been used as a prominent model for the study of estrogen receptor-positive breast cancer cells. In MCF-7 cells Dex is able to prevent the cytotoxic effect of TNF-α, and the anti-apoptotic proteins IAP1, IAP2 and XIAP have been postulated as effector molecules [16]. However, and despite extensive studies, the molecular mechanisms of this protection are just beginning to be described. On this respect, the role played by anti-apoptotic routes others than the one regulated by IAP proteins in the Dex protection from TNF-α cytotoxicity has not been analyzed. Also, TNF-α stimulation does not only activate cell death pathways, but survival ones too. In consequence, it can be assumed that the balance between pro- and anti-apoptotic regulators defines the apoptotic threshold of a cell. The anti-apoptotic effect of TNF-α requires the activation of PI3K and NF-κB and, as active participants of survival routes, these proteins could participate in the Dex protection against TNF-α cytotoxicity. Thus, we analyzed their participation in Dex mediated-protection against TNF-α cytotoxicity.

It has been suggested that the scarcity of breast tumor derived cell lines has led to the apparition of several sub-lines, evidenced by different results obtained for the evaluation of related phenomena [28], including their susceptibility to TNF-α induced apoptosis [29].

This led us to corroborate the ability of TNF-α to induce cell death and to evaluate the protection mediated by Dex in our cell system. As previously reported, TNF-α treatment induced cell death in a dose and time dependent fashion and co-incubation with Dex protected MCF-7 cells against TNF-α-induced cell death.

We have found that in MCF7 and ZR-70-35 human mammary tumor cells the protective effect of Dex was compromised in the presence of 2.5 μM of Bay-117082, a pharmacological inhibitor of NF-κB activation (data not shown). This result correlates with those observed in Figure 3D, where protection is lost in cells expressing the dominant negative form of IkBα, thus providing further support to the notion that Dex protection requires NF-κB activation. Furthermore, the use of the inhibitor of NF-κB lead to a marked decrease in c-IAP1 cellular content in ZR-70-35 cells (data not shown). While c-IAP1 could not be detected in TNF-α-treated cells, in the presence of TNF-α + Dex c-IAP1 content returned to control levels. This behavior reproduced the results presented in figures 4C and 4D, and documents the correlation between Dex protection and c-IAP1 cellular content.

In our system the Akt phosphorylation level was not affected by Dex treatment in the presence or absence of TNF-α. Besides, transfection of a dominant negative mutant of PI3K (ΔP85) in MCF-7 cells did not affect Dex protection, suggesting that the PI3K/Akt pathway is not involved in Dex protection against TNF-α. NF-κB activation through PI3K/Akt has been a controversial issue due to cell type variations [30,31]. Although in some cells Akt acts upstream of NF-κB [24,32,27], we found that NF-κB activation is completely independent of Akt function.

In our cell system Dex did not modify the NF-κB activation in the presence or absence of TNF-α. However, a non-degradable IκBα mutant protein (dnIκBα), which prevents NF-κB nuclear translocation; completely blocked Dex protection against TNF-α induced cell death. In the absence of Dex, dnIκBα expression increased the susceptibility to TNF-α-induced death. These results suggest that the TNF-α-dependent NF-κB activation participates in the protection conferred by Dex. Furthermore, in dnIkBα transfectant MCF-7 cells, the susceptibility to TNF-α cytotoxicity correlated with the level of expression of the IκBα mutant form, suggesting a threshold for the protective action of NF-κB activation.

NF-κB regulates the expression of a great number of genes, including several antiapoptotic gene products such as members of the Bcl-2 family [33] and the inhibitor of apoptosis proteins XIAP, c-IAP1 and c-IAP2 [19]. Interestingly, NF-κB regulates XIAP [34] and cIAP1 promoters [35]. Thus, we analyzed the effects of interfering with NF-κB signaling pathway during Dex protection on XIAP and c-IAP1 protein content. We detected that, as previously reported [16], TNF-α-induced apoptosis in MCF-7 cells correlated with downregulation of XIAP and c-IAP1 proteins, postulated as effectors of the protective effect against TNF-α-mediated cytotoxicity. However, only the expression level of c-IAP1 correlated with the protective effect of Dex: In cells expressing dnIκBα stimulated with TNF-α (i) the protein level of this antiapoptotic factor was lower than in parental cells and correlated with an increased cell death and, (ii) in parental MCF-7 cells Dex treatment correlated with a slower rate of decrease of the anti-apoptotic factors content.

While estrogen dependence in mammary tumor cells is being extensively studied due to its clinical importance, the dependency on GC has received less attention. The protection conferred by Dex against TNF-mediated cytotoxicity has been extensively analyzed in MCF-7 cells and, interestingly, this synthetic GC has also been reported to confer protection against pharmacological mediators of cell death [36]. In contrast, GCs have been reported to interfere with proliferation in MCF7, ZR-75-1, Con-8 and MDA-MB-231 mammary tumor cells [37,38]. At present, GC therapy is not included in patients with mammary tumors, although no comparative study has been performed to discard its efficacy. Whether or not the antiproliferative effect of natural or synthetic GCs is related to the protection against TNF-mediated cytotoxicity remains to be determined.

Also, IAPs belong to a diverse group of proteins which modulate the apoptotic pathways by binding to caspases and inhibiting their proteolitic activity [39]. In addition to this well characterized anti-apoptotic effect, some IAP isoforms have been reported to interfere with apoptosis through caspase-inhibition independent mechanisms. Expression of IAPs in MCF-7 cells in response to Dex has been previously described and is suggestive of the anti-apoptotic protection against TNF-mediated cytotoxicity. Nevertheless, the contribution of IAP expression to this protective effect remains to be shown by specific interference with IAPs expression, possibly through iRNA technology. Without this kind of experiments, it is difficult to establish the relative contribution of IAP expression to the protective effect of Dex.

## Conclusion

We conclude that the protective effect of Dex is dependent on TNF-α-mediated activation of NF-κB, and it seems likely that the NF-κB-dependent gene expression of antiapoptotic proteins is strenghtened by Dex treatment, probably through the GC receptor. This protection appears to be independent of the PI3K pathway. Moreover, GC-receptor activation through Dex has been reported to induce the expression of different anti-apoptotic gene products, including c-IAP1 and XIAP [19,40], serum and GC-inducible protein kinase one (SGK-1), and mitogen activated protein kinase phosphatase one (MKP-1) [41]. On this respect, we found the existence of a suggestive correlation between susceptibility against TNF-α-induced cell death and the diminished c-IAP expression in the absence of NF-κB translocation. Although the interaction among glucocorticoids and cytokines is often cell type specific and depends on the physiologic context of the cell, our data point towards the NF-κB system as a potential therapeutic target in the combat against some hormone-dependent forms of mammary cancer.

## Methods

### Materials

Dexamethasone and human recombinant TNF-α were obtained from Sigma-Aldrich (St Louis, MO, USA) and R&D Systems, Inc (Minneapolis, MN, USA), respectively. Cell culture media and sera were obtained from InVitrogen Life Technologies (San Diego, CA, USA). The polyclonal rabbit antibodies against Akt and against phosphorylated Akt were from Cell Signalling Technology, Inc (Beverly, MA, USA). IκB goat polyclonal antibody was from Santa Cruz Biotechnology, Inc. (Santa Cruz, CA, USA). XIAP and c-IAP1 polyclonal antibodies were from R&D Systems Inc. Protease inhibitor cocktail tablets were from Boehringer Manheim (East Sussex, UK). Secondary antibodies were from Pierce Biotechnology Inc. (Rockford, IL, USA) (anti-rabbit IgG) and Zymed Laboratories (Carlsbad, CA, USA) (anti-mouse and anti-goat IgG). The Super Signal Chemiluminescent substrate was from Pierce Biotechnology Inc. *Escherichia coli DH5α *strain was from Gibco BRL (Paisley, UK). Plasmids containing the cDNAs for negative mutant phosphoinositide-3-kinase (Δp85) [42] and constitutively active mutant IκB alpha, Ser 32/36 -Ala (dnIκBα) [43] were a gift from Dr. Masato Kasuga (The Second Department of Internal Medicine, Kobe University School of Medicine) and Dr. David V. Goeddel and Dr. Dean W. Ballard (Howard Hughes Medical Institute and Department of Microbiology and Immunology, Vanderbilt University School of Medicine, Nashville, TN), respectively. The cDNAs from Δp85 and dnIκBα were sub-cloned into the expression vector pCNLX-GFP under the control of the cytomegalovirus (CMV) promoter.

### Cell culture

MCF-7 cells were purchased from the American Type Culture Collection (ATCC, Manassas, VA, USA) and were maintained in RPMI-1640 medium supplemented with 10% (v v^-1^) foetal calf serum (FCS), 100 U ml^-1 ^penicillin, 100 μg ml^-1 ^streptomycin and 2 mM L-glutamine, and incubated at 37°C in a humidified atmosphere with 5% CO_2_.

### Transfection assays

*Escherichia coli DH5α *cells were transformed with either of the plasmids, dnIκBα or Δp85, using plasmidic DNA obtained with a Wizard extraction kit (Quiagen GmbH, Germany). MCF-7 parental cells were stably transfected with 200 μg μl^-1 ^of each of the plasmids using the calcium phosphate transfection system from Gibco BRL according to manufacturer's instructions. After 24 h, transfected cells were selected in G418-containing medium (0.8 mg ml^-1^) (Sigma-Aldrich). Transfection efficiency was evaluated by GFP fluorescence. Single clones of stably transfected cells, isolated by limiting dilution in 96-well plates (Nalgene Nunc International, Rochester, NY, USA), were transferred to individual plates and cultured in medium containing 0.5 mg ml^-1 ^G418. Expression of the Δp85 and dnIκBα proteins were assessed by Western blot analysis using anti-PI3K and anti-IκB specific antibodies. For MCF-7 cells expressing the Δp85 protein, three independent clones were used throughout this study: A6, A8 and A10. For the dnIκBα expressing cells two independent clones were used: C1 and C8. A clone of an MCF-7 cell transfected with the empty expression vector pCNLX-GFP was used as control and it always provided the same results as did the parental cell line.

### Cytotoxic Assays

For all the cytotoxic assays 1 × 10^4 ^cells were plated per well in 48-well plates and cultured for 24 h. In a first set of experiments, cell cultures were treated with increasing concentrations (2–10 ng ml^-1^) of TNF-α for different periods of time. In subsequent experiments, cell cultures were either co-incubated with 10 ng ml^-1 ^TNF-α and 10 μM Dex, or 10 ng ml^-1 ^TNF-α and Dex vehicle (ethanol) for 24 h. Cell number was assessed indirectly by cell staining with crystal violet.

### Western blot analysis

Cells (1 × 10^5^) were plated in 60 cm^2 ^Petri dishes, cultured for 24 h and incubated with 10 ng ml^-1 ^TNF-α, 10 μM Dex or both, for different periods of time. Cell cultures were washed in ice cold Tris-buffered saline (TBS, 50 mM Tris-HCl, 150 mM NaCl, pH 7.5) and lysed for 20 min on ice chilled lysis buffer (50 mM Tris, 0.5% Nonidet P-40, 120 mM NaCl, 200 μM Na_3_VO_4_, 100 mM NaF, 1 mM PMSF, pH 8.0, added with 1 protease inhibitor cocktail tablet). Protein extracts were clarified by centrifugation (14,000 × g, 15 min 4°C) and protein content measured by Bradford (BioRad, Hercules, CA, USA). Equal amounts of total protein (40 μg) were subjected to 10% SDS/PAGE followed by transfer onto nitrocellulose membranes followed by Western blot analysis and visualized with the Super Signal system (Pierce). Membranes were incubated with antibodies against Akt protein, phosphorylated Akt protein, IκB, XIAP or c-IAP1 and detected with the respective species-specific secondary HRP-conjugated antibodies.

### Preparation of nuclear protein extracts

Nuclear protein extracts were obtained from cell cultures after the indicated treatments. Briefly, cells were washed and scraped into ice-cold phosphate-buffered saline (PBS). Cells were pelleted at 4°C and then frozen in ethanol-dry ice for 1 min. Cells were immediately resuspended in 100 μl of buffer A (10 mM HEPES, 10 mM KCl, 1.5mM MgCl_2_, 1mM DTT, pH 7.9) and incubated 10 min at 4°C. Nuclei were microcentrifuged, resuspended in 30 μl of buffer B (20 mM HEPES, 400 mM NaCl, 1.5 mM MgCl_2, _0.2 mM EDTA, 25% glycerol, 1 mM DTT, 0.5 mM PMSF pH 7.9) and incubated on ice for 30 min. Nuclei suspension was microcentrifugated for 20 min, and then the supernatant (nuclear protein extract) was diluted with 30 μl of HDKE buffer (20 mM HEPES, 50 mM KCL, 25% glycerol, 0.2 mM EDTA, 1 mM DTT, 0.5 mM PMSF, pH 7.9), and aliquots stored at -70°C. Protein concentrations of the nuclear extracts were determined using the Bradford-based BioRad protein assay.

### Electrophoretic mobility shift assay (EMSA)

Binding assays were performed in a final volume of 20 μl containing nuclear protein extract (10 μg), buffer HDKE, 1mM DTT, 10 μg BSA, 1μg poly(dI-dC) (Amersham Biosciences, Germany) and 1 μl of end-labelled (γ-^32 ^P) NF-κB oligonucleotide at 5000 cpm μl^-1 ^(AGTTGAGGGGACTTTCCCAGG, Santa Cruz Biotechnology, Inc). Reactions were incubated for 20 min at room temperature. Protein-DNA complexes were separated from free oligonucleotide on a 5% nondenaturing polyacrylamide/Tris borate EDTA gel. The gels were dried and analyzed in a Molecular Dynamics "Storm" Phosphoimager using the Image Quant software (Molecular Dynamics, San Jose, CA, USA).

### Data analysis

All experiments were performed in triplicates and repeated at least three independent times. All statistical analyses were performed using a nonparametric Kruskal-Wallis test and corroborated with a two-way ANOVA test with Bonferroni posttests for individual values. A p < 0.05 was considered statistically significant.

## List of abbreviations

DD, death domain; dnIKBα, non-degradable IkBα mutant protein; Dex, dexamethasone; Δp85, negative mutant phosphoinositide-3-kinase; EMSA, electrophoretic mobility shif assay; FADD, Fas associated DD; GC, glucocorticoids; GFP, green fluorescent protein; GR, glucocorticoid receptor; HRP, horseradish peroxidase; IAP, inhibitory anti-apoptotic protein; MKP-1, mitogen activated protein kinase phosphatase one; NF-κB, nuclear factor-kappa B; NIK, NF-kB inducing kinase; pAkt, phosphorilated Akt; PI3K, phosphatidyl inositol 3 kinase; SGK-1, serum and GC-inducible protein kinase one; TBS, tris-buffered saline; TNF-α, tumor necrosis factor-alpha; TNF-R1, TNF-α receptor type 1; TRADD, TNF-R associated DD.

## Authors' contributions

CAM carried out the cell culture, molecular biology (cloning and sequencing of the mutants) and biochemistry studies (EMSA and Western blot analysis), participated in statistical analyses, in the design of the study and drafted the manuscript. CRM participated in biochemistry studies. EC, SM, participated in result analyses and manuscript preparation, LC participated in construction of de pCNLX-GFP plasmid, JV participated in transfection assays. AZ, conceived of the study, its design and coordination. All authors participated in discussion and approval of the final form of the manuscript.
